# Berberine-Loaded Carboxylmethyl Chitosan Nanoparticles Ameliorate DSS-Induced Colitis and Remodel Gut Microbiota in Mice

**DOI:** 10.3389/fphar.2021.644387

**Published:** 2021-04-20

**Authors:** Luqing Zhao, Xueying Du, Jiaxin Tian, Xiuhong Kang, Yuxin Li, Wenlin Dai, Danyan Li, Shengsheng Zhang, Chao Li

**Affiliations:** ^1^ Digestive Disease Center, Beijing Hospital of Traditional Chinese Medicine, Capital Medical University, Beijing, China; ^2^ State Key Laboratory of Chemical Resource Engineering, Beijing University of Chemical Technology, Beijing, China; ^3^ Center for Applied Statistics, Institute of Statistics and Big Data, Renmin University of China, Beijing, China

**Keywords:** berberine, nanoparticles, colitis, drug delivery, gut microbiota

## Abstract

Inflammatory bowel disease (IBD) is a refractory disorder characterized by chronic and recurrent inflammation. The progression and pathogenesis of IBD is closely related to oxidative stress and irregularly high concentrations of reactive oxygen species (ROS). A new oxidation-responsive nano prodrug was constructed from a phenylboronic esters-modified carboxylmethyl chitosan (OC-B) conjugated with berberine (BBR) that degrades selectively in response to ROS. The optimized micelles exhibited well-controlled physiochemical properties and stability in a physiological environment. OC-B-BBR micelles could effectively encapsulate the anti-inflammatory drug berberine and exhibit ideal H_2_O_2_-triggered release behavior as confirmed by *in vitro* drug loading and release studies. The *in vivo* anti-inflammatory effect and regulation of gut microbiota caused by it were explored in mice with colitis induced by dextran sodium sulfate (DSS). The results showed that OC-B-BBR significantly ameliorated colitis symptoms and colon damage by regulating the expression levels of IL-6 and remodeling gut microbiota. In summary, this study exhibited a novel BBR-loaded Carboxylmethyl Chitosan nano delivery system which may represent a promising approach for improving IBD treatment.

## Introduction

Inflammatory bowel disease (IBD) is a refractory gastrointestinal disorder characterized by chronic and recurrent inflammation. Ulcerative colitis (UC) and Crohn's disease (CD) are the two main forms of IBD (Kaser et al., 2010). IBD has a high incidence in the United States (more than 1 million) and in Western Europe (2.5 million), and has evolved into a widespread disease with increasing prevalence all over the world (Kaplan, 2015). The pathogenesis of IBD is still not fully understood, but it is mainly considered to be associated with environmental factors, host genetic susceptibility, changes in intestinal flora and intestinal epithelial barrier dysfunction, and other factors (Zundler and Neurath, 2015). Oxidative stress plays an essential role in the pathogenesis and progression of IBD (Tian et al., 2017), and leads to excessive ROS accumulation. Biopsies from patients with IBD demonstrate abnormally high levels of ROS at the site of the lesion, with mucosal ROS concentrations increasing from 10- to 100-fold (Simmonds et al., 1992; Lih-Brody et al., 1996).

Although there is no clear evidence of a relationship between IBD and mortality, there is no doubt that IBD has an adverse impact on the quality of life of patients. Many drugs are available for the treatment of UC including 5-Aminosalicylic acid, steroids, immunosuppressant, probiotics, biological agents, herbal medicines, and so on. Ulcerative colitis can be cured with FMT in patients who do not respond to other more accessible treatments. However, IBD is difficult to cure permanently at present. Taking medicine inevitably brings about many adverse reactions and consumes a lot of medical resources (Shivaji et al., 2019; Wilke et al., 2020).

Berberine (BBR) is an isoquinoline alkaloid, often used as an antidiarrheal, derived from the rhizome of *Coptis chinensis* (“Huang-Lian” in Chinese) of the Ranunculaceae family. Recently, BBR and its derivatives have been examined for use in IBD treatment (Massironi et al., 2013; Wakuda et al., 2013). It is worth noting that BBR may alleviate intestinal inflammation through different mechanisms. It seems to be related to the regulation of the Treg/Th17 balance by modifying gut microbiota (Cui et al., 2018). In addition, BBR could identify bitter taste receptors on intestinal Tuft cells and activate IL-25-ILC2-IL-13 immune pathway to impair damaged intestinal tract by promoting differentiation of intestinal stem cells (Xiong et al., 2021). There is also a broad space to improve the efficacy and bioavailability of BBR. For example, its absolute bioavailability has been reported to be less than 1% (Chae et al., 2008; Chen et al., 2011) and the plasma level of BBR is very low, although the significant pharmacological effects of BBR have been observed in clinic (Hua et al., 2007).

**FIGURE 1:**
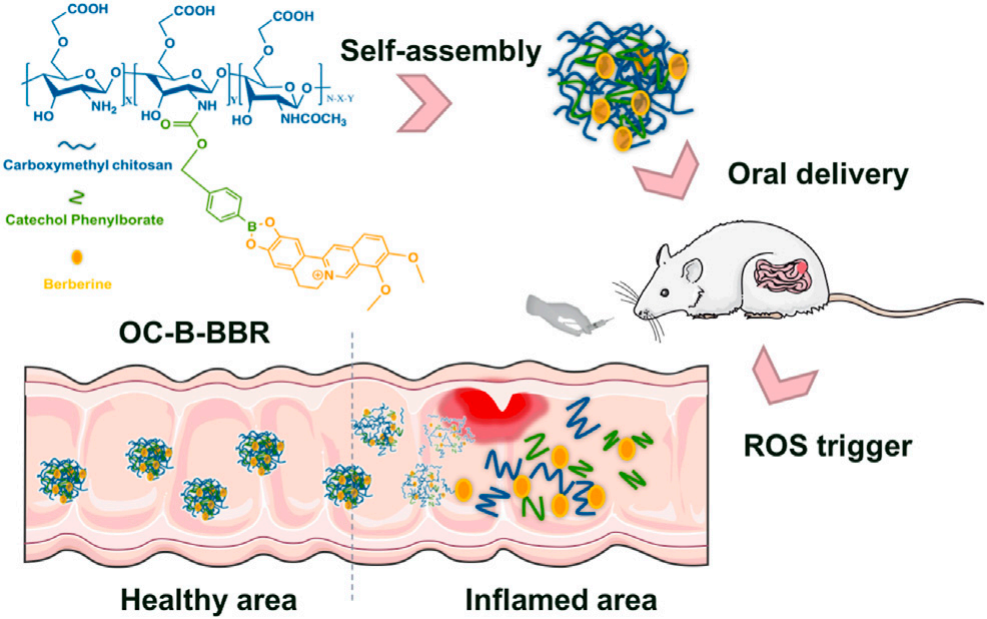
The schematic presentation of OC-B-BBR prodrug micelles structure and their inflamed-selective anti-inflammatory drug delivery by ROS-triggered mechanism.

A targeted drug delivery system can ensure that the drug is released only at the intestinal inflammation site instead of healthy tissue. Targeting and selectivity are achieved by the abnormally high concentration of ROS in the inflammation site (Wilson et al., 2010; Zhang et al., 2016). Because the nanosized targeted delivery system will mainly accumulate in the inflamed part of the intestine, it is considered to be an excellent idea for the treatment of IBD (Lamprecht et al., 2001). Santos’ group reported a nano-in-micro composite to achieve an oxidation-responsive delivery of rifaximin (RIF) for IBD treatment. RIF mediates changes in epithelial cell physiology and reduces bacterial attachment and internalization, and also antagonized the effects of tumor necrosis factor-α on intestinal epithelial cells by activating pregnane X receptor, which inhibits nuclear factor-κB-mediated proinflammatory mediators and induces detoxification genes. RIF-loaded nanoparticles have been prepared by phenylboronic esters-modified dextran (OxiDEX). Under physiologically relevant H_2_O_2_ concentrations, the nanoparticles exhibited a high degree of H_2_O_2_-responsive degradation ability and controlled drug release (Bertoni et al., 2018). Nanoparticles are likely to adjust the properties of drugs, such as stability, solubility, and release ability, and their surface is easily modified to introduce targeting ligands, and even adjust surface characteristics, including surface charge and adhesion properties. Consequently, we here proposed a functional prodrug micelle as an inflammation-targeted drug, which was comprised of BBR covalent linked to biocompatible carboxylmethyl chitosan by aryl boronic ester as responsive linker. This nanosystem was adopted so that chitosan-based prodrug micelles could effectively deliver berberine to inflamed tissue by ROS responsive mechanism, improving its bioavailability in the specific site (Figure 1). Compared with the RIF-loaded delivery system, BBR in the current system was covalently linked to the carrier by unique catechol group, which can precisely achieve ROS-responsive and prevent the premature release of drugs in the delivery process. The synthesis and physiochemical properties of polymeric OC-B-BBR were explored to optimize micelle structure. The ability of the system to release berberine in physiological and simulated ROS overexpression medium was also investigated. *In vivo* anti-inflammatory effect and regulation of gut microbiota by it were explored in mice with colitis induced by dextran sodium sulfate (DSS), which showed features in common with ulcerative colitis in humans.

## Materials and Methods

### Materials

Carboxylmethyl chitosan (OC, Mw = 37 kDa, degree of deacetylation = 88.7%) was purchased from Macklin. Berberine (BBR) was purchased from Saen Chemical Technology Co., Ltd. The Spectra/Por 1 dialysis membrane (MWCO: 3,500) was purchased from Spectrum Laboratories. All the other reagents and solvents were provided by Beijing Chemical Reagent Co., Ltd. and used without further purification. A Bruker AV-400 nuclear magnetic resonance spectroscope was used to record all NMR spectra at 400 MHz in CDCl_3_ (unless otherwise specified). An Agilent 6540 UHD Q-TOF MS (analyzed ions up to m/z 6,000) equipped with a gas nebulizer probe was used to record data of High-resolution mass spectra (HRMS). DSS was purchased from MP Biomedicals Co., Ltd. TNF-α and IL-6 enzyme-linked immunosorbent assay (ELISA) kits were purchased from Wuhan servicebio technology Co., Ltd. TGF-β and IL-23 ELISA kits were purchased from Multisciences (LIANKE) Biotech Co., Ltd.

### Preparation of OC-B-BBR Micelles

Phloroglucinol (2.5 g, 19.8 mmol) was dissolved in 60% H_2_SO_4_ (50 ml) and stirred at room temperature until the raw materials were completely dissolved to form a colorless solution. Berberine (2.5 g, 7.4 mmol) was added to the above solution, and the mixture was stirred at 95°C for 15 min. Then the reaction was transferred to the saturated NaCl solution and stirred at room temperature for 2 h. The solution was filtered, and the filter cake was dissolved with methanol. The resulted solution was concentrated *in vacuo* and washed by ethyl acetate for twice. The solution was then filtered again, upon which the compound demethyleneberberine was obtained as a dark red solid (yield 90%). 1H-NMR (400Mz, DMSO-*d*_6_): *δ*/ppm = 3.105–3.136 (t, 2H, - CH_2_), 4.070 (s, 3H, - CH_3_), 4.095 (s, 3H, - CH_3_), 4.898–4.929 (t, 2H, - CH_2_), 6.884 (s, H, Ph-H), 7.557 (s, H, Ph-H), 8.052–8.075 (d, H, Ph-H), 8.173–8.196 (d, H, Ph-H), 8.768 (s, H, Ph-H), 9.432 (bs, H, -OH), 9.857 (s, H, Ph-H), 10.246 (bs, H, -OH).

We dissolved carboxymethyl chitosan (OC, 128 mg, 0.24 mmol) and 0.2 ml of tetramethylguanidine in 15 ml deionized water, then stirred the mixture for 30 min 4-nitrophenyl [4-(4,4,5,5-tetramethyl-1,3,2-dioxaborolan-2-yl)phenyl]methyl ester (NBC, 0.2 g, 0.501 mmol) was dissolved in 5 ml THF, which was added dropwise to the above mixture at 25°C. After the reaction was finished, the solution was dialyzed against water for 2 days. The product OC-B was obtained by lyophilization.

The OC-B-BBR micelles were prepared using dialysis method. In brief, OC-B (20 mg) was dissolved in 8.4 ml deionized water and saturated NaHCO_3_ was added to adjust pH = 8. Then 3 ml DMA was added to the above solution with stirring for 10 min at room temperature. 10 mg of demethyleneberberine dissolved in methanol (2 ml) were slowly added to the mixture, then stirred for 8 h at 25°C. The resulting solution was dialyzed against water/methanol (1,000 : 2, v/v) for 2 days (molecular weight cut off 3,500 Da) to remove by-products, and then lyophilized to obtain a dry reddish brown flocculent product of OC-B-BBR micelles (60 mg).

### Characterization of Physiochemical Properties

Gel permeation chromatography (GPC) (TDK 302, Viscotek, USA) was used to determine the molecular weight distribution of the analytes, in which the mobile phase was water. A Zetasizer Nano instrument (Zetaplus, Brookhaven, USA) and a HeeNe laser (633 nm) were used to measure the particle size of micelles by dynamic light scattering (DLS) to collect optical measurements. All analytes were suspended in pH 7.4 PBS at a concentration of 1 mg mL^−1^ in DLS measurement. S-4700 cold field emission scanning electron microscope (SEM, Hitachi, Japan) was used to analyze the surface morphology of polymeric products and obtain SEM images. Double-sided tape was used to mount the sample for SEM to the metal post, and a thin layer of gold was sputtered under vacuum. For zeta potential measurement, a Nano-ZS ZEN3600 particle sizer (Malvern Instruments) was used.

The encapsulation efficiency (EE) and loading capacity (LC) of OC-B-BBR micelle were depicted as Eqs. 1 and 2, respectively.

$$\text{EE}\left(\%\right)=\frac{\text{weight of Barberine in micelle}}{\text{weight of Barberine feed}}\times 100\%$$ (1)

$$\text{LC}\left(\%\right)=\frac{\text{weight of encapsulated Berberine}}{\text{total weight of micelle}}\times 100\%$$ (2)

### *In Vitro* Drug Release of Micelles

A dialysis membrane was used to evaluate the release of OC-B-BBR micelles under sink conditions. For simulated ROS released study, H_2_O_2_ with different concentrations was added into phosphate-buffer solution (PBS, pH 7.4) as the release media. In brief, 2 mg of OC-B-BBR micelles (DS = 13.1%) were kept in a dialysis bag (MWCO: 3.5 kDa), sealed and placed in 200 ml of release medium, and continuously shook at 100 rpm at 37°C. 1 ml of buffer solution was collected at various time intervals, and 1 ml of fresh medium was added in time after each collection. The cumulative amounts of berberine were determined by Waters Alliance HPLC (UV-detector, λ = 360 nm, C-18 column, eluent: 0.2% phosphoric acid in water: acetonitrile (36:64, v/v), flow rate: 1.0 ml min^−1^).

### Animal Experiments and Dosage Information

Animal experiments were designed according to ARRIVE 2.0 Guideline. C57BL/6 J mice aged 6–8 weeks were obtained from the SPF (Beijing) Biotechnology Co., Ltd (permission number: SCXK (jing) 2019–0,010). The mice were cultured under standard conditions (temperature of 20–26°C, relative humidity of 40–70%, light-dark cycle of 12/12 h, clean bedding, free access to water, and standard dry pellet diet). After a week of adaptive feeding, the mice were randomly divided into four groups, with eight mice in each group. The groups were as follows: 1) Control group, continually fed water alone for 10 days; 2) DSS group, colitis was induced with 3% DSS, which was added to their drinking water for 6 days, on the fourth day, started to use normal saline for gavage for 7 days; 3) OC-B-BBR group, colitis was induced with 3% DSS, which was added to their drinking water for 6 days, on the fourth day, started to use nano-berberine for gavage (30 mg kg^−1^·d^−1^) for 7 days; 4) Mesalazine group, colitis was induced with 3% DSS for 6 days, on the fourth day, started to use mesalazine for gavage (100 mg kg^−1^) for 7 days.

Animal studies were performed according to the NIH Guide for the Care and Use of Laboratory Animals and were approved by Animal Care and Use Committee of Beijing Hospital of traditional Chinese Medicine, Capital Medical University.

### Disease Activity Index (DAI)

The body weight, stool viscidity, and fecal occult blood were observed daily, and the DAI (Yan et al., 2018) of the mice were measured, including body weight loss (the percentage of weight loss relative to the initial body weight, where 0 score = none, 1 score = 1%–5%, 2 score = 5%–10%, 3 score = 10%–20%, 4 score => 20%), stool viscidity (0 score = normal, 2 score = loose, 4 score = diarrhea), and fecal occult blood (0 score = no blood, 1 score = +, 2 score = ++, 3 score = +++, 4 score = gross bleeding). DAI = (body mass index + stool viscidity + bleeding)/3.

### Sample Collection and Measurement

Colons and spleens were excised from sacrificed mice, and then the length of colons and weight of the spleens were measured. The spleen index = wet weight of the spleen (mg)/the bodyweight (g). Feces were collected for 16 S ribosomal RNA (16SrRNA) analysis. Portions of the colon were fixed in 10% formalin and then embedded in paraffin sections for hematoxylin-eosin (H&E) staining. A portion of that colon was store at -80°Cfor ELISA analysis.

### Intestinal Mucosal Injury Index Analysis

The colons were dissected to observe the damages on intestinal mucosa. The extent of the damage was graded by colonic mucosa damage index (CMDI) (Yan et al., 2018), scored as follows: 0, no injury to the colonic mucosa; 1, the surface of intestinal mucosa is smooth, no erosion or ulcer, but with mild hyperemia and edema; 2, has congestion and edema, the mucosa is coarse and granular, with erosion or intestinal adhesion; 3, necrosis and ulcers appeared on the surface of intestinal mucosa, which also has high congestion and edema (the maximum longitudinal diameter of the ulcer is shorter than 1.0 cm), moreover, the intestinal wall surface has necrosis and inflammation or the hyperplasia of intestinal wall; and 4, the maximum longitudinal diameter of ulcer is longer than 1.0 cm, or with total intestinal wall necrosis more severe than 3 points.

### Histological Analysis

H&E stained sections of colonic tissue was determined by two independent, blinded investigators following a scoring system for inflammation-associated histological changes in the colon (Wirtz et al., 2007). The scoring system for inflammation-associated histological changes in the colon was: 0, No evidence of inflammation; 1, Low level of inflammation with scattered infiltrating mononuclear cells (1–2 foci); 2, Moderate inflammation with multiple foci; 3, High level of inflammation with increased vascular density and marked wall thickening; and 4, Maximal severity of inflammation with transmural leukocyte infiltration and loss of goblet cells.

### ELISA Analysis

Levels of TNF-α (purchased from Wuhan servicebio technology Co., Ltd.), IL-6 (purchased from Wuhan servicebio technology Co., Ltd.), TGF-β (purchased from Multisciences (LIANKE) Biotech Co., Ltd.), and IL-23 (purchased from Multisciences (LIANKE) Biotech Co., Ltd.) in colon tissue were quantified using ELISA kits according to the instructions.

### 16SrNA Analysis

Magpure stool DNA KF kit B (Magen, China) was used to extract genomic DNA from feces. 30ng of qualified genomic DNA samples and corresponding fusion primers were used to configure the PCR reaction system. The v3-v4 region of 16 S rRNA of genomic DNA was amplified by setting the PCR reaction parameters. The PCR products were purified with agencourt ampure XP magnetic beads, dissolved in elusion buffer, labeled, and completed the establishment of the library. Agilent 2,100 Bioanalyzer was used to detect the fragment range and concentration of the library. According to the size of inserted fragments, hiseq platform was selected for sequencing. After getting off the machine, the data were filtered, and the reads were spliced into tags through the overlap relationship between reads. Under the given similarity, tags were aggregated into out, and the OTU representative sequences were compared with the database by RDP classifer (V2.2) software for species annotation, and the confidence threshold was set to 0.6. Based on OTU and annotation results, species complexity analysis, species diversity analysis, and correlation analysis were carried out.

### Statistical Analysis

Multiple groups were compared by one-way analysis of variance (ANOVA). *t*-test or Mann-Whitney *U*-test was used to compare the two groups. Data were expressed as mean ± standard deviation (SD). *p* < 0.05 was considered statistically significant.

## Results

### Design and Synthesis of Berberine Nanomicelle

Carboxylmethyl chitosan was chosen as a carrier to improve solubility and biocompatibility of micelles for drug delivery. Stimuli-responsive phenyl borate ester as a linker conjugated berberine to carboxylmethyl chitosan by effective aminolysis reaction. The synthesis route of the nanocarrier OC-B-BBR was depicted in Supplementary Scheme S1. Initially, NBC was prepared according to previous reports (Jourden and Cohen, 2010; Chung et al., 2018), as a key intermediate for linking glycol chitosan and berberine. Phenyl boronate moiety was linked to 2-NH_2_ on carboxylmethyl chitosan by aminolysis, and subsequently borate ester was then easily hydrolyzed under alkaline conditions to give OC-B. A simple strategy was proposed to conjugate berberine by its unique catechol structure. The exposed boronic acid group can spontaneously react with catechol in water to form stable borate ester (OC-B-BBR). The successful synthesis of OC-B-BBR nanocarrier was confirmed by 1H NMR spectra. The characterization of OC and OC-B was also presented as controls to better assign the proton signals of berberine in OC-B-BBR (Supplementary Figures S1–S3). The amphiphilic OC-B-BBR conjugates readily formed self-assembled micelles in an aqueous environment. In mild excess ROS environment, the stimuli-responsive borate ester was broken to release berberine molecules.

### Physicochemical Properties of Micelles

The physiochemical properties of nanocarriers should be carefully considered to achieve target special delivery of the particles. The primary concerns, including degree of substitute (DS), particle size, encapsulation efficiency (EE), and loading capacity (LC), were determined as shown in Table 1. The feed ratio of the reaction was changed to adjust the DS of berberine, which can greatly affect the self-assembly behavior and hydrophilic-lipophilic balance of the micelles. Phenyl boronic ester was firstly grafted to OC with different DSs ranged from 10.08 to 26.02%, and the mean diameters of the micelles, measured by DLS, were in the 120–140 nm range. The poor solubility of berberine and reactivity of phenolic hydroxyl (alcoholic hydroxyl was more likely to react with phenylboric acid), to a large extent, limited the increase of DS. The optimized OC-B-BBR (DS = 13.6%) was used to evaluate the subsequent drug release and *in vivo* anti-inflammatory efficacy.

**TABLE 1:** Physiochemical properties of OC, OC-NBC, and OC-B-BBR.

Samples	M_w_ (Da) ± SD	M_n_ (Da) ± SD	M_w_/M_n_ ± SD	Particle size (nm) ± SD	PDI^b^ ± SD	EE (%)^c^ ± SD	LC (%)^d^ ± SD
OC	37,695 ± 1,348	17,127 ± 1,123	2.2 ± 0.2	-^a^	-	-	-
OC-NBC	54,985 ± 2,350	22,982 ± 2,140	2.4 ± 0.3	222.7 ± 26.4	0.42 ± 0.12	-	-
OC-NBC-BBR	17,049 ± 929	14,497 ± 827	1.2 ± 0.1	230.2 ± 18.1	0.22 ± 0.09	67.5 ± 4.4	13.1 ± 1.6

^a^ no data.

^b^ polydispersity index.

^c^ Encapsulation efficiency, see section Materials and Methods for calculation.

^d^ Loading capacity, see section Materials and Methods for calculation.

**FIGURE 2:**
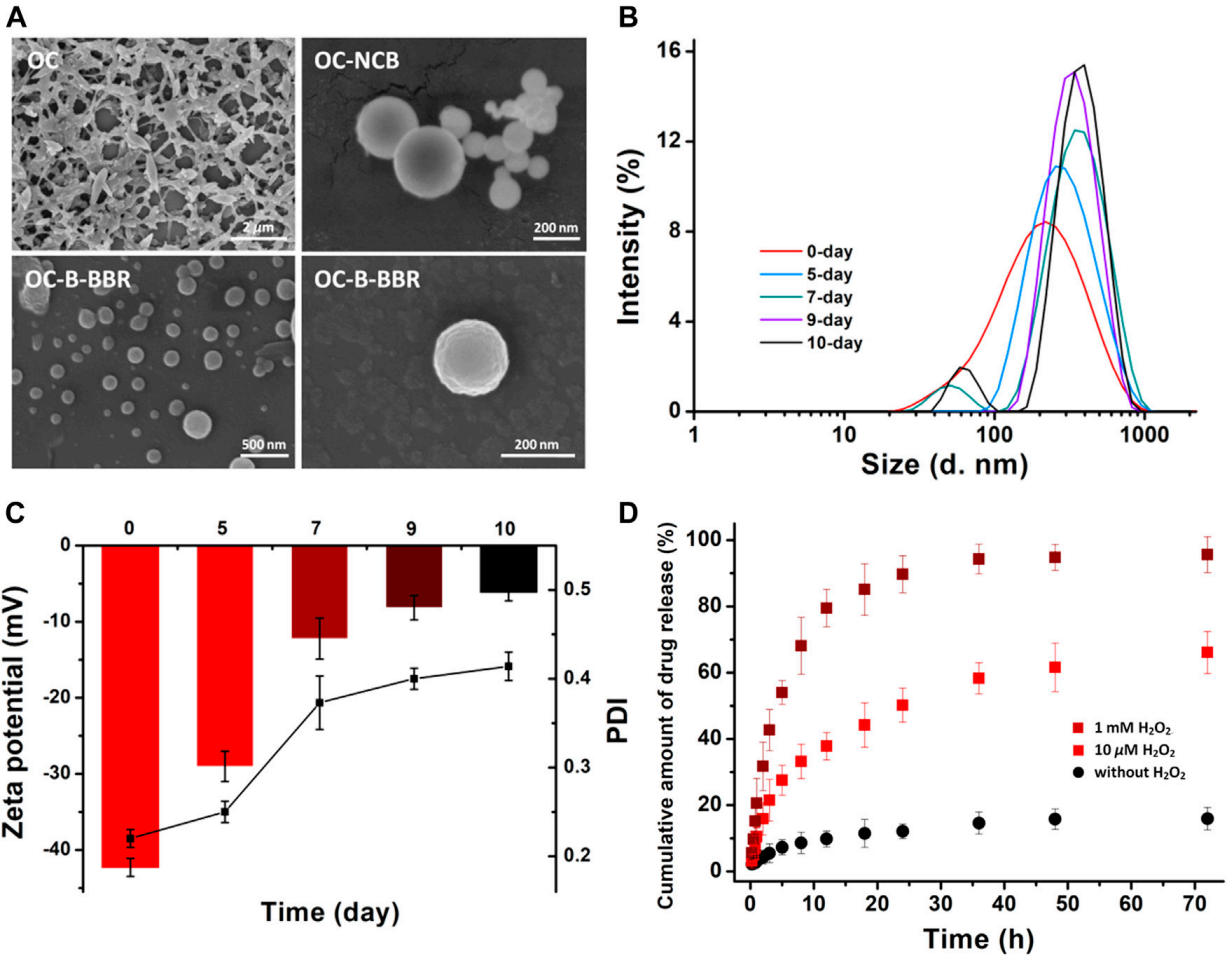
**(A)** SEM images of the different polymers. **(B)** The changes in OC-B-BBR nanoparticle size in pH 7.4 medium. **(C)** The changes in surface charges and PDI **(D)** *In vitro* drug release profiles of berberine. The OC-B-BBR micelle was incubated in pH = 7.4 buffer without H_2_O_2_ (black dot), 10 *μ*M H_2_O_2_ (red square), and 1 mM H_2_O_2_ (wine square), as measured through HPLC.

The nanoscale morphology of the obtained structure was shown by electron microscope images. As shown in Figure 2A by SEM measurement, OC and blank OC-B were spherical nanoparticles (about 100 nm) and cross-linked. The micelles of OC-B-BBR maintained good microsphere morphology, as shown in Figure 2A in different scales. The average particle size of the nanoparticles was about 130 nm, and slightly larger than the carrier OC-B. The change trends of particle size measured by DLS were basically the same as that measured by SEM. For the specific data of particle size, the DLS results were slightly larger than the SEM results. This was mainly because the structure was in a hydrated state during DLS measurement, which made the size of the structure larger.

Subsequently, the stabilities of OC-B-BBR micelles were monitored on the basis of variation in sizes and surface charges. In pH = 7.4 medium, the micelles showed insignificant changes in size and a negligible decrease in zeta potential in 5 days (Figures 2B,C), suggesting the good stability of the micelles that was essential for a prolonged blood half-life *in vivo*.

### *In Vitro* Drug Release Profile

The drug release profiles in physiological and simulated ROS environment were explored. As shown in Figure 2D without H_2_O_2_, less than 20% berberine was released in 72 h incubation at pH 7.4, indicating that the micelles had satisfactory stability around physiological conditions. When 10 *μ*M H_2_O_2_ was added in the micelles system, 50% of berberine was collected in 24 h incubation, indicating the ROS-sensitivity of OC-B-BBR micelles. At least 65% of the drugs were released over the tested time. The same assays were performed again with 1 mM H_2_O_2_ incubation, and the rate of berberine release sharply increased. More than 90% of the drugs can be released in 24 h incubation, showing the sensitivity to excess ROS. The sustained and thorough release indicated that the nanomicelles have a favorable response ability in inflamed tissues with high levels of ROS, which promoted drug delivery efficacy.

### OC-B-BBR Showed a Potential Role in Ameliorating the Colitis Induced by DSS in Mice

**FIGURE 3:**
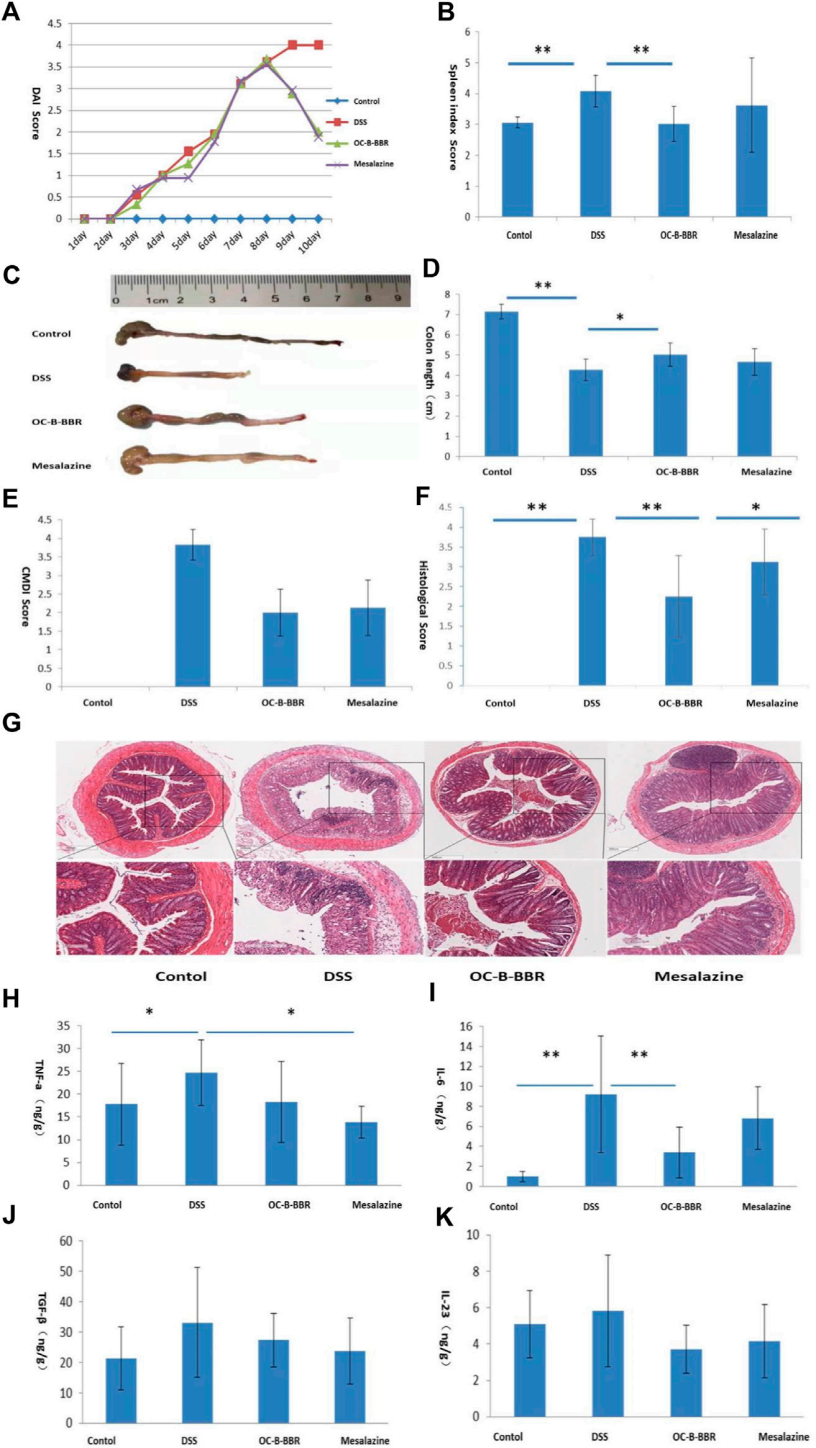
OC-B-BBR ameliorated DSS-induced colitis in mice. Results represent mean ± SD; n = 8, *p < 0.05; **p < 0.01. **(A)** disease activity indexes (DAI); **(B)** Spleen index; **(C)** Colon picture; **(D)** Colon length; **(E)** Colonic mucosa damage index (CMDI) score; **(F)** Histological score; **(G)** H&E staining of sections displayed colonic tissue damage and leukocyte infiltration, ✕4 and ✕10; **(H)** TNF-α; **(I)** IL-6; **(G)** TGF-β; **(K)** IL-23.

To study the effect of OC-B-BBR in colitis, five parameters, DAI, colon length, spleen index, CMDI score, and histological score, were evaluated. Mice in the DSS group presented more severe colitis than mice in the OC-B-BBR group, as evidenced by a significant increasing of DAI (Figure 3A) and spleen index (*p* < 0.01) (Figure 3B), and shortening of the colon (*p* < 0.05) (Figures 3C,D). The damage on intestinal mucosa by visual inspection in the DSS group presented significantly higher congestion and edema, more serious erosion or intestinal adhesion, bigger ulcers, and higher CDMI score (*p* < 0.01) (Figure 3E) compared with the OC-B-BBR group. Similarly, the inflammatory cell infiltration and histological score of H&E staining sections of colon tissue in the DSS group were significantly higher compared with the OC-B-BBR group (*p* < 0.01) (Figures 3F,G). There were no significant differences between the OC-B-BBR and mesalazine group in the DAI, colon length, spleen index, or CMDI score. The histological scores of the OC-B-BBR group were significantly lower than that of the mesalazine group (*p* < 0.05). Above all, OC-B-BBR could effectively ameliorate DSS-induced colitis in mice and may have potential advantages over mesalazine.

### OC-B-BBR Suppressed the Secretion of Some Inflammatory Cytokines in DSS-Induced Mice

Excessive production of proinflammatory cytokines lead to the progression and exacerbation of colitis. To understand the anti-inflammatory effect of OC-B-BBR, we measured the levels of proinflammatory cytokines in the colon homogenates by ELISA. The results showed that the levels of TNF-α and IL-6 were significantly increased after DSS-induced (*p* < 0.05 or *p* < 0.01). The increase of IL-6 was significantly reduced by OC-B-BBR treatment (*p* < 0.01), while the increase of TNF-α was significantly reduced by mesalazine treatment (*p* < 0.05). There were no significant differences of TGF-β and IL-23 in the four groups (Figures 3H–K).

### OC-B-BBR Modified Gut Microbiota

**FIGURE 4:**
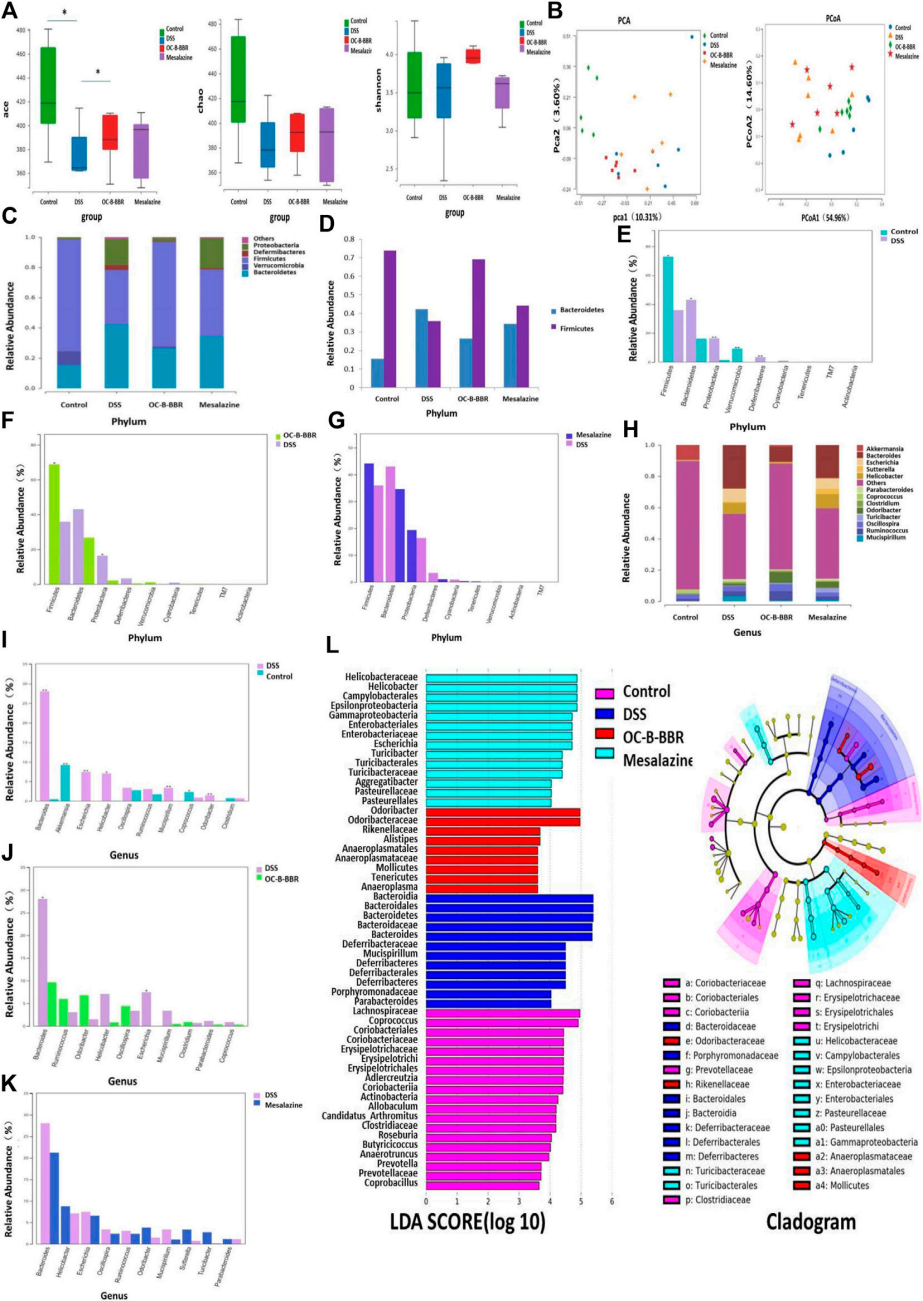
**(A)** *α*-Diversity estimated by ACE, Chao, and Shannon indexs,*p < 0.05; **(B)** PCA score and PCoA score plots; **(C)** Gut microbiota composition at phylum level in each group; **(D)** Firmicutes/Bacteroidetes ratio at phylum level in each group; **(E)** The proportions of key species at phylum level, Normal group vs DSS group; **(F)** The proportions of key species at phylum level, OC-B-BBR group vs DSS group; **(G)** The proportions of key species at phylum level, Mesalazine group vs DSS group; **(H)** Gut microbiota composition at genus level in each group; **(I)** The proportions of key species at genus level, Normal group vs DSS group; **(J)** The proportions of key species at genus level, OC-B-BBR group vs DSS group; **(K)** The proportions of key species at genus level, Mesalazine group vs DSS group; **(L)** LDA score and LEfSe taxonomic cladogram.

α-diversity analysis reflected the richness and diversity of microbial communities, including a series of statistical analysis indexes. The Chao and ACE indexes are used to estimate the microbial richness, while Shannon index is used to estimate the microbial diversity. The ACE index of DSS group was significantly lower than that of the normal group, of which the OC-B-BBR group and mesalazine group were significantly increased (*p* < 0.05) (Figure 4A). Although the Chao index did not increase significantly, the increase in species richness was demonstrated to some extent (Figure 4A). Shannon index showed no significant difference among the four groups (Figure 4A). No apparent clustering was observed in principal component analysis (PCA) (Figure 4B) or principal coordinate analysis (PCoA) (Figure 4B) among the normal group, DSS group, OC-B-BBR group, or mesalazine group.

At phylum level, the compositions of gut microbiota in each group were shown in Figure 4C. Compared with the normal group, the relative abundance of Bacteroidetes, Deferribacteres, and Proteobacteria in the DSS group increased significantly, while the relative abundance of Firmicutes and Verrucomicrobia decreased significantly (*p* < 0.05 or *p* < 0.01) (Figures 4D,E). Compared with the DSS group, the relative abundance of Firmicutes increased significantly in the OC-B-BBR group, while the relative abundance of Proteobacteria decreased (*p* < 0.05) (Figure 4F). There was no significant difference between the DSS group and mesalazine group (Figure 4G).

At genus level, the compositions of gut microbiota in each group were shown in Figure 4H. Compared with the normal group, the relative abundance of *Bacteroides*, *Escherichia*, *Helicobacter*, and so on increased significantly in the DSS group, while the relative abundance of Akkermansia and Coprococcus decreased significantly (*p* < 0.05 or *p* < 0.01) (Figure 4I). Compared with DSS group, the relative abundance of *Bacteroides* and *Escherichia* decreased significantly in the OC-B-BBR group (*p* < 0.05) (Figure 4J). There was no significant difference between the DSS group and mesalazine group (Figure 4K).

LEfSe linear discriminant analysis (LDA) was used to discriminate the significant different species (LDA>4, *p* < 0.05). The higher the LDA score, the greater effect of the relative abundance of species on the difference. There were significant differences in the composition of key species among the four groups. A LEfSe taxonomic cladogram represented key bacterial alterations. Different colors of purple, blue, red, and green respectively represent the NC, model, OC-B-BBR, and mesalazine groups. Each small circle at different taxonomic levels represents a taxon at that level, and the diameter of the small circle is proportional to the relative abundance (Figure 4L).

## Discussion

Chinese herbal medicine treatment of inflammatory bowel disease has a long history in China and around the world (Zhao et al., 2017). However, due to the low content of active ingredients and oral bioavailability, the further improvement of the curative effect of Chinese herbal medicine is limited. The development of targeted drug delivery based on nano technology presents a new approach for natural drugs extracted from Chinese herbal medicine in the treatment of IBD.

Chitosan is a natural polymer of living organisms, which provides a basis for the construction of functional polymer biomaterials with biological properties and unique physicochemical properties, biocompatibility, and biodegradability. In particular, through the controlled functionalization of some simple borate parts, we can obtain an intelligent system for specific site drug delivery with certain required response characteristics, such as ROS response (Maji et al., 2015). Herein, based on ROS responsiveness, we designed a new type of chitosan nanocarrier to achieve targeted drug delivery to inflammation sites. The ROS responsive group, which can effectively deliver the encapsulated BBR to the inflammatory site by ROS-triggered release in the microenvironment of oxidative stress, was formed by the self-assembly of amphiphilic carboxymethyl chitosan and phenylborate side groups. The selectivity of phenylborate as the linker was mainly due to its ROS responsiveness and biocompatibility, as well as convenient conjugation with drugs *via* catechol moiety. Many natural anti-inflammatory drugs contained catechol, such as quercetin and rutin. Thus, the current design provided a realistic and general strategy for constructing catechol-based responsive nanodrug delivery system.

Dynamic light scattering (DLS) was used to evaluate the size and polydispersity index (PDI) of the OC-B-BBR micelles. The hydrodynamic average size was about 220 nm, with a relatively similar particle size distribution of 0.22 (Table 1). However, in the case of the empty nanocarrier OC-B, the detected particle size distribution was slightly wider at 0.42, due to the weak hydrophobicity in the core without drugs. This result was consistent with the observation on SEM. In the subsequent stability tests, the relatively intact micelles were obtained in a few days by DLS and zeta potential analysis, which provided a vital guarantee for stable drug delivery before reaching the inflammatory site.

Here our results showed that OC-B-BBR alleviated DSS-induced colitis in mice. Compared with the DSS group, after OC-B-BBR treatment the DAI score and spleen index was decreased, the colon length was increased, and the damage to the colon (congestion, edema, erosion, ulcer or inflammatory cell infiltration etc) was reversed. A particular concern was that histological scores were significantly lower in the OC-B-BBR group than in the mesalazine group, while there were no significant differences between the two groups on other indicators. This showed that for histological healing OC-B-BBR may have had potential advantages over mesalazine. Histological deep healing is the highest goal of clinical treatment for IBD patients. A recent systematic review and meta-analysis revealed that patients who achieved endoscopic and histological remission have a significantly lower risk of clinical relapse than patients who achieved clinical remission (Yoon et al., 2020). The proinflammatory cytokines levels correlate with the severity of colitis. Our results showed that OC-B-BBR treatment inhibited the release of IL-6, while mesalazine treatment inhibited the release of TNF-α. It suggests that OC-B-BBR and mesalazine may exert anti-inflammatory effects through different pathways.

The pathogenesis of IBD is complicated and not clear. It is now accepted that a complex interplay of genetic and environmental factors and gut microbiota lead to abnormal immune responses and chronic colitis (Nishida et al., 2018). Compared with healthy people, IBD patients had less bacteria with anti-inflammatory capacities and more bacteria with inflammatory capacities (Peterson et al., 2008). The most recognized changes were a decrease in the diversity of the intestinal microbial community, a decrease in abundance of Firmicutes, and increases in abundance of Proteobacteria and Bacteroidetes (Manichanh et al., 2006; Walker et al., 2011). Our results showed that OC-B-BBR increased the community richness of gut microbiota decreased by DSS induction. At phylum and genus level, compared with the DSS group, the relative abundance of Firmicutes was increased, while the relative abundance of Proteobacteria, *Bacteroides*, and *Escherichia* was decreased in the OC-B-BBR group. In the DSS group, the ratio of Firmicutes and Bacteroidetes was inverted, and OC-B-BBR treatment was shown to restore its normal trend. OC-B-BBR treatment shifted the microbiome toward a “healthy” phenotype. The relative abundance of species in the mesalazine group were not significantly different from the DSS group. OC-B-BBR may attenuate DSS-induced colitis by modulating the composition of bacterial communities. In future studies, we need to explore the mechanism of OC-B-BBR inhibiting inflammation by regulating gut microbiota.

## Conclusions

In summary, BBR was conjugated to carboxylmethyl chitosan by aryl boronic ester, giving a potential ROS responsive for an effective delivery of drugs to the inflammatory tissue. OC-B-BBR as carboxylmethyl chitosan nanomicelles were prepared and characterized, which ameliorates DSS-induced colitis and remodels gut microbiota. The novel natural drug nano delivery system may represent a promising approach for improving IBD treatment.

## Data Availability

The original contributions presented in the study are included in the article/Supplementary Material, further inquiries can be directed to the corresponding authors.
