# Supplementary material for: Berberine-Loaded Carboxylmethyl Chitosan Nanoparticles Ameliorate DSS-Induced Colitis and Remodel Gut Microbiota in Mice
Source: Front Pharmacol. 2021 Apr 20;12:644387. doi: 10.3389/fphar.2021.644387 (PMC8093821; doi:10.3389/fphar.2021.644387)
Supplement: Supplementary file 1 [file datasheet1.doc]

# Supplementary Materials

**Berberine-loaded** **Carboxylmethyl Chitosan Nanoparticles ameliorate DSS-induced colitis and remodel gut microbiota in mice**

**Luqing Zhao^1^, Xueying Du^2^, Jiaxin Tian^2^, Xiuhong Kang^1^, Yuxin Li^1^, Wenlin Dai^3^, Danyan Li^1^, Shengsheng Zhang^1*^, Chao Li^2*^**

^1^Digestive Disease Center, Beijing Hospital of Traditional Chinese Medicine, Capital Medical University, Beijing, 100010, P. R. China

^2^State Key Laboratory of Chemical Resource Engineering, Beijing University of Chemical Technology, Beijing 100029, P. R. China

^3^Center for Applied Statistics, Institute of Statistics and Big Data, Renmin University of China, Beijing 100872, P. R. China

*** Correspondence:**

Shengsheng Zhang

[zhangshengsheng@bjzhongyi.com](mailto:zhangshengsheng@bjzhongyi.com)

Chao Li
lichao@mail.buct.edu.cn


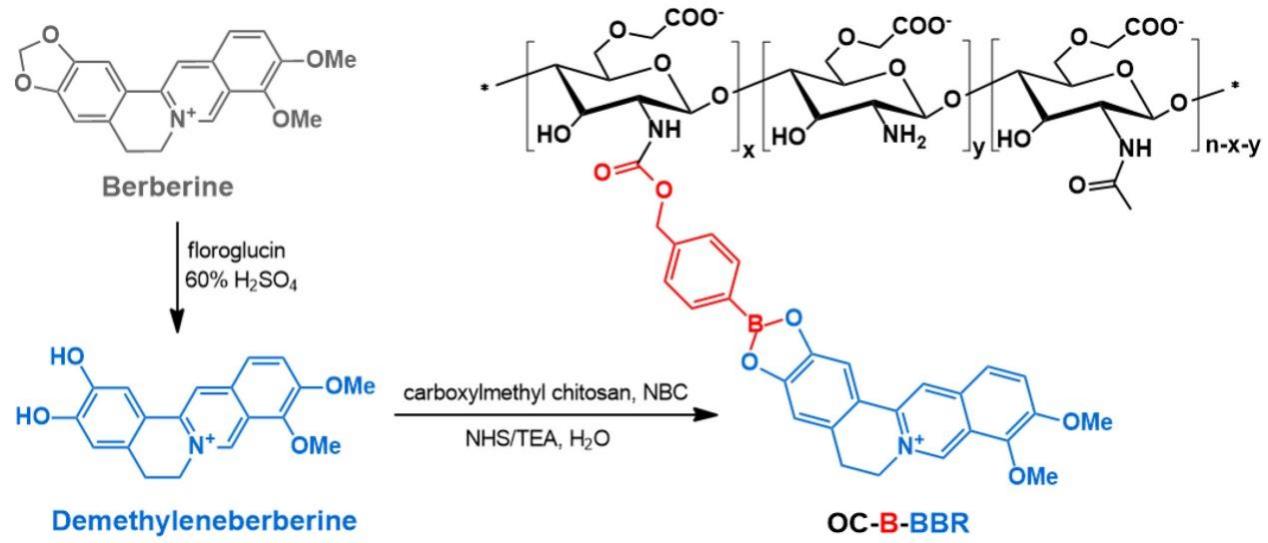


**Scheme S1**. The structure and synthetic route of **OC-B-BBR** nanoparticle.


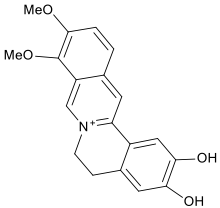


22

24

2

1

7

10

18

11

12

15

b

a

a

b

7

10

1 2

18

15

11

12

22 24

**Figure S1.** ^1^H-NMR of spectrum demethyleneberberine


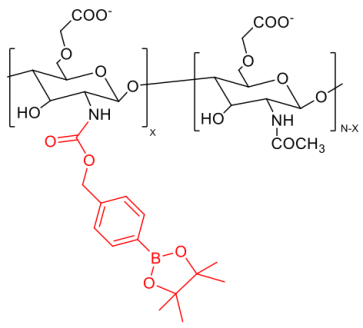


3’

5‘

4’

1

2

3

4

5

6

7

3’

4’

5‘

1

3-6

2

7

**Figure S2.** ^1^H-NMR spectrum of **OC-NBC**


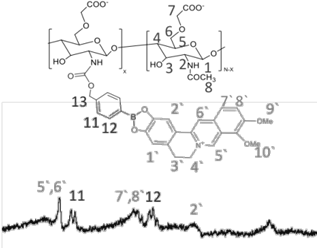


1，7，4’

3，5，6，9‘，10’

2，3‘

8

4

**Figure S3.** ^1^H-NMR spectrum of **OC-B-BBR**
